# Cdc42 improve SARS-CoV-2 spike protein-induced cellular senescence through activating of Wnt/β-Catenin signaling pathway

**DOI:** 10.3389/fcimb.2024.1449423

**Published:** 2024-11-04

**Authors:** Chunmei Nong, Zhenzhen Wu, Chan Yang, Wei Xu, Linyi Luo, Jianping Zhou, Lihan Shen, Yinghua Chen, Yaoqin Yuan, Guodong Hu

**Affiliations:** ^1^ The First School of Clinical Medicine, Southern Medical University, Guangzhou, Guangdong, China; ^2^ Department of Respiratory and Critical Care Medicine, The Tenth Affiliated Hospital of Southern Medical University, Dongguan, Guangdong, China; ^3^ Department of Respiratory and Critical Care Medicine, Institute of Respiratory and Critical Care Medicine, Dongguan, Guangdong, China; ^4^ Guangdong Provincial Key Laboratory of New Drug Screening, School of Pharmaceutical Sciences, Southern Medical University, Guangzhou, Guangdong, China; ^5^ Intensive Care Unit, The Tenth Affiliated Hospital of Southern Medical University, Dongguan, Guangdong, China; ^6^ Department of Thoracic Surgery, The Tenth Affiliated Hospital of Southern Medical University, Dongguan, Guangdong, China; ^7^ Dongguan People’s Hospital Biobank, Clinical Research Center, The Tenth Affiliated Hospital of Southern Medical University, Dongguan, Guangdong, China; ^8^ Guangdong Provincial Key Laboratory of Construction and Detection in Tissue Engineering, School of Basic Medical Sciences, Southern Medical University, Guangzhou, Guangdong, China

**Keywords:** SARS-CoV-2, spike, senescence, Cdc42, β-catenin

## Abstract

**Introduction:**

SARS-CoV-2 infection drove senescent cells and the senescence-associated phenotypes were reported playing roles in disease progression, which contributes to severe COVID-19 and related sequelae. Cdc42 is involved in the regulation of cellular senescence. This study, aimed to investigate the mechanism of the SARS-CoV-2 spike protein regulating cellular senescence through Cdc42.

**Methods:**

K18-hACE2 mice were infected with SARS-CoV-2 Omicron BA.4 or stimulated with spike protein through the airway, the senescent cells and Cdc42 expression in lung tissue were detected. Overexpression of spike protein or exogenous incubation of spike protein was used to simulate the induction of cellular senescence by spike protein. Mechanistic insights into the role of Cdc42 were mainly explored using Western Blot and qRT-PCR.

**Results:**

Spike protein, SARS-CoV-2 infection related, accelerates cell aging by upregulating Cdc42 expression, which furtherly activated the Wnt/β-catenin signaling pathway. Conversely, treatment with ML141 in animal models, a Cdc42 inhibitor, reduced cellular senescence and ameliorated lung injury and inflammation. These results suggest that the upregulation of Cdc42 by the SARS-CoV-2 spike protein induces cellular senescence and enhances β-catenin nuclear translocation.

**Discussion:**

This study provides insights into the mechanisms underlying cellular senescence induced by the SARS-CoV-2 spike protein, offering potential strategies to mitigate the inflammatory response and complications associated with COVID-19 in both the acute and long-term phases.

## 1 Introduction

Since its emergence as a global pandemic in December 2019, SARS-CoV-2 has infected over 700 million people worldwide, leading to more than 7 million deaths as of February 2024 (World Health Organization, 2024). Notably, a significant proportion of patients continue to experience persistent symptoms or related sequelae even after the virus has cleared, including systemic fatigue, chest pain, respiratory dysfunction, nervous system, and cognitive impairments (Ballering et al., 2022; Ceban et al., 2022; Davis et al., 2023; Mandal et al., 2021; Mo et al., 2020). Consequently, a deeper understanding of the biological responses to SARS-CoV-2 infection, particularly the persistent manifestations, is urgently required.

Aging is recognized as a significant risk factor for severe COVID-19, with older individuals being more susceptible to severe disease manifestations. This heightened susceptibility is primarily attributed to age-related impairments in pulmonary function and immune responses within the aging lung (Bartleson et al., 2021; Schneider et al., 2021). Cellular senescence, initially observed in normal diploid cells, ceasing proliferation after a limited number of replications and divisions (Hayflick and Moorhead, 1961), is considered a response to various stressors, including exposure to toxic substances, hypoxia, mitochondrial dysfunction, activation of oncogenes, and viral infections (Gorgoulis et al., 2019). Cellular senescence represents an irreversible state of cell-cycle arrest accompanied by alterations in transcriptional, epigenetic, morphological, secretory properties, and metabolic capacity (Wiley and Campisi, 2021). While senescence serves as a beneficial mechanism for tumor suppression and wound healing (Campisi, 2001; Demaria et al., 2014; Munoz-Espin and Serrano, 2014), the aberrant accumulation of senescent cells can contribute to an inflammatory microenvironment, chronic tissue damage, and the onset and progression of chronic diseases such as chronic obstructive pulmonary disease and pulmonary fibrosis (Araya et al., 2019; Barnes et al., 2019; Guan et al., 2022). A principal hazard associated with these cells is their ability to secrete a range of bioactive substances, including proinflammatory factors, chemokines, growth factors, and matrix metalloproteinases, collectively referred to as senescence-associated secretory phenotypes (SASP) (Gorgoulis et al., 2019; Rodier and Campisi, 2011). The release of these factors contributes to the development of acute or chronic inflammation and potentially regulates immune responses. Furthermore, SASP can induce secondary senescence in neighboring healthy and proliferative cells, through paracrine signaling while activating immune surveillance (Acosta et al., 2013; Coppe et al., 2008).

SARS-CoV-2 induces cellular senescence through various mechanisms, a phenomenon known as virus-induced senescence (VIS) (Meyer et al., 2021; Tripathi et al., 2021). Bulk and single-cell transcriptomic analyses further support the concept that SARS-CoV-2 triggered senescence drives pathology in COVID-19 and the lungs of patients with severe COVID-19 showed higher levels of p16^INK4a^ positive cells compared with that in individuals with other lung diseases (Schmitt et al., 2022). Senescence is a universal host cell response to SARS-CoV-2 stress. VIS cells aggravate the inflammatory response by secreting a plethora of SASP factors, many of which are bonafide NF-κB targets. Besides, only VIS cells show activation of the cGAS–STING pathway and are involved in driving SASP-mediated interferon responses, which indicates that virus-induced senescence is a driver and therapeutic target in COVID-19 (Lee et al., 2021). Given that older individuals already have a high prevalence of senescent cells, exposure to SARS-CoV-2 may increase this burden, potentially leading to an abnormal release of SASP and resulting in a cascade of inflammatory factors that culminate in an inflammatory storm. This inflammatory storm may be a primary reason why older individuals are more susceptible to developing severe illness from COVID-19.

SARS-CoV-2 S1 protein exacerbates the SASP of human senescent cells, leading to an active inflammatory response in severe patients. Furthermore, anti-aging drugs targeting senescent cells have been found to reduce mortality rates in infected mice (Weiss and Murdoch, 2020). Moreover, SARS-CoV-2 triggers senescence in infected cells, upregulating the expression of ACE2 receptors, thereby increasing the likelihood of infection and establishing a vicious cycle (Camell et al., 2021; Duarte et al., 2021). Even after viral clearance, the persistence of senescent cells in lung tissue, evidenced by the increased number of cells expressing p16 and p21, may contribute to post-acute COVID-19 syndrome (Lipskaia et al., 2022). These findings suggest that senescent cells play a role in the pathogenesis of COVID-19, highlighting the importance of reducing cellular senescence for the treatment of COVID-19 and the prevention of its long-term effects.

Cell division cycle protein 42 (Cdc42) is a small GTPase belonging to the Rho family, which plays a pivotal role in various fundamental cellular processes, including the reorganization of the actin cytoskeleton, cell polarity, and growth (Cerione, 2004; Fu et al., 2022; Mosaddeghzadeh and Ahmadian, 2021). Aberrant activation of Cdc42 has been implicated in several pathological conditions, including carcinogenesis and neurodegenerative diseases (Sinha and Yang, 2008). In addition to its involvement in these pathological states, recent research has highlighted the critical role of Cdc42 in cellular senescence. Inhibiting Cdc42 activity can restore the regenerative potential of senescent intestinal stem cells. Furthermore, Cdc42 is implicated in the senescence process of hematopoietic stem cells and mesenchymal stem cells (Florian et al., 2013; Nalapareddy et al., 2021; Umbayev et al., 2018). In studies involving aged mice, a specific inhibitor of Cdc42 called Casin has been utilized to reduce chronic inflammation levels and extend the average lifespan(Florian et al., 2020).

The removal of senescent cells, either through genetic or pharmacological means, can reportedly delay the onset of age-related inflammatory diseases in aged mice (Baker et al., 2016; Di Micco et al., 2021). Building upon the observed relationship between Cdc42 and cellular senescence, our study aimed to explore the potential of targeting Cdc42 as a therapeutic and preventive approach for COVID-19 and its associated complications.

## 2 Materials and methods

### 2.1 Animals

All animal experiments and protocols conducted in this study were approved by the National Institutional Animal Care and Medical Ethics Committee of Southern Medical University. Male-specific pathogen-free (SPF) K18-hACE2 mice, aged 8−10 weeks, were obtained from Gempharmatech company (Jiangsu, China). Prior to the experiment, all mice were acclimated in cages for five days. The animal model was generated using two approaches: infection with SARS-CoV-2 Omicron BA.4 and stimulation with SARS-CoV-2 spike protein via the trachea.

#### 2.1.1 Infection of SARS-CoV-2 Omicron BA.4

The mice were allocated randomly into control and BA.4 groups (n=6 each). Mice in the control group were intranasally inoculated with 50 μL of PBS. Conversely, mice in the BA.4 group were intranasally inoculated with 10^5^ plaque-forming units (PFU) of SARS-CoV-2 Omicron BA.4, prediluted in 50 μL of PBS. On the fourth day following inoculation, all mice were euthanized, and organ tissues were collected for histopathological analyses. The experimental procedures were conducted in accordance with approved guidelines under Biosafety Level 3 (BSL-3) conditions.

#### 2.1.2 Stimulation of SARS-CoV-2 spike protein

The mice were segregated randomly into control, SARS-CoV-2 ancestral spike, and SARS-CoV-2 omicron spike groups (n=6 each). K18-hACE2 mice were stimulated via the trachea with 5 μg spike protein, prediluted in 40 μL of PBS. The control group received an equivalent volume of PBS similarly. For mice undergoing ML141 treatment, ML141 (8 mg/kg) was administered intraperitoneally 1 h prior to SARS-CoV-2 spike protein stimulation. This regimen was administered once daily for five consecutive days during the same time period. On the sixth day, the mice were euthanized for further analysis.

### 2.2 Hematoxylin and Eosin (H&E) staining

Lung tissues from experimental mice were fixed in 4% paraformaldehyde for 48 h and embedded in paraffin. Sections (5μm thick) were stained with H&E and examined under a light microscope (Nikon, Japan) to assess histopathological alterations.

### 2.3 Immunohistochemistry assay

Hydrated sections were separately incubated in citrate buffer (pH 6.0), followed by exposure to 3% H2O2. A blocking solution with 10% goat serum was applied at room temperature for 1 h. The sections were then incubated overnight at 4°C with primary antibodies, including anti-p16 (1:200, Affinity Biosciences, OH, USA), anti-p21 (1:200, ABMART, China), and anti-Cdc42 (1:250, Abcam, UK). Subsequently, the sections were incubated with HRP-labeled goat anti-rabbit IgG or anti-mouse IgG secondary antibodies (both from ZSGB-BIO, Beijing, China) for 1 h at 37°C, and immunoreactivity was detected using DAB staining (Solarbio, Beijing, China). Quantitative analysis of related indicators was performed using ImageJ 1.8.0.

### 2.4 qRT-PCR

Total RNA was extracted using the FastPure Cell/Tissue Total RNA Isolation Kit V2 (RC112-01, Vazyme, Nanjing, China), and reverse transcription was performed using the PrimeScript™ RT Master Mix (RR036A, Takara Biomedical Technology, Beijing, China) according to the manufacturer’s protocol. For quantitative real-time polymerase chain reaction (qRT-PCR), SYBR Green Real-time PCR Master Mix (Q711-02, Vazyme, Nanjing, China) was used. The sequences used are shown in **Table 1**.

**Table 1:** Primer sequences for qRT-PCR.

Gene	Forward primer 5′-3′	Reverse primer 5′-3′
p16	CTTCCTGGACACGCTGGT	GGGATGTCTGAGGGACCTT
p21	TCGCTCAGGGGAGCAGGCTGAA	CTCGCGCTTCCAGGACTGCAGGCT
IL-1β	ATGATGGCTTATTACAGTGGCAA	GTCGGAGATTCGTAGCTGGA
IL-6	ACTCACCTCTTCAGAACGAATTG	CCATCTTTGGAAGGTTCAGGTTG
TNF-α	CCTCTCTCTAATCAGCCCTCTG	GAGGACCTGGGAGTAGATGAG
IL-8	GAAGTTTTTGAAGAGGGCTGAGA	GCCCTTGGCCTCAATTTTGC
GAPDH	CACATGGCCTCCAAGGAGTAA	TGAGGGTCTCTCTCTTCCTCTTGT
m-TNF-α	CAGGCGGTGCCTATGTCTC	CGATCACCCCGAAGTTCAGTAG
m-IFN-γ	GCCACGGCACAGTCATTGA	TGCTGATGGCCTGATTGTCTT
m-IL-17	TTTAACTCCCTTGGCGCAAAA	CTTTCCCTCCGCATTGACAC
m-IL-6	CTGCAAGAGACTTCCATCCAG	AGTGGTATAGACAGGTCTGTTGG
m-IL-1β	GAAATGCCACCTTTTGACAGTG	TGGATGCTCTCATCAGGACAG
m-β-actin	GGCTGTATTCCCCTCCATCG	CCAGTTGGTAACAATGCCATGT

### 2.5 Western blot analysis

According to the provided proportions, RIPA Lysis Buffer (ES-8148-100ml, ECOTOP SCIENTIFIC, Guangzhou, China), Protease Inhibitor Cocktail (20124ES03, Yeasen, Shanghai, China), and Phosphatase Inhibitor Cocktail (20109ES05, Yeasen, Shanghai, China) were prepared to extract the protein. Approximately 20 μg of proteins were separated by SDS-PAGE and then transferred to polyvinyl difluoride (PVDF) membranes (ISEQ00010, Merck Millipore, Darmstadt, Germany). Primary antibodies, including anti-p16 (AF0228-50μL, Affinity Biosciences, OH, USA), anti-p21 (TD6423S, Abmart, China), anti-Cdc42 (10155-1-AP, Proteintech, USA), anti-β-catenin (51067-2-AP, Proteintech, USA), and anti-GAPDH (FD0063, Hangzhou Fude Biological Technology, Hangzhou, China) were used. The next day, Goat Anti-Rabbit HRP IgG (FDR007, Hangzhou Fude Biological Technology, Hangzhou, China) and Goat Anti-Mouse HRP IgG (Hangzhou Fude Biological Technology, Hangzhou, China) were used for room-temperature incubation.

### 2.6 Cell culture, plasmids, and transfection

HEK-293T cells stably expressing human ACE2 (ACE2/293T) were previously described (Yang et al., 2021). Similarly, we generated A549 cells that stably express human ACE2 receptors using the same method. The cells were cultured in Dulbecco’s Modified Eagle’s Medium (DMEM) or RPMI-1640 medium supplemented with 10% fetal bovine serum (FBS) and 1% penicillin-streptomycin. The cell cultures were maintained in a humidified atmosphere containing 5% CO_2_ at 37°C. The plasmid encoding the SARS-CoV-2 S protein (pcDNA3.1-SARS-CoV-2 S) was described in a previous study (Yang et al., 2021). Additionally, our laboratory constructed the pcDNA3.1-Spike-omicron plasmid and maintained an empty vector, pcDNA3.1. To perform transfections, the cells were cultured overnight in 6-well plates and transfected with the desired plasmid (pcDNA-SARS-CoV-2 S, pcDNA3.1-Spike-omicron) or the empty vector (2.5μg/well). Liofectamine 3000 (L3000001, ThermoFisher, USA) was used for transfection following the manufacturer’s instructions. The corresponding assays or experiments were conducted 72 hours after transfection.

### 2.7 Senescence-associated β-galactosidase expression

The senescence-associated β-galactosidase (SA-β-Gal) activity in ACE2/A549 cells overexpressing spike proteins was assessed using a cellular senescence staining kit (C0602, Beyotime Biotechnology, China) following the manufacturer’s instructions. The staining procedure allows the visualization of blue-colored cells indicative of senescent cells.

### 2.8 Statistical analysis

Data are presented as mean ± SD. Statistical comparisons were performed using one-way analysis of variance (ANOVA) and unpaired Student’s t-tests, utilizing GraphPad Prism 9. Significance thresholds were set at *p<0.05, **p<0.01, and ***p<0.001.

## 3 Results

### 3.1 SARS-CoV-2 spike protein promotes cellular senescence

**Figure 1:**
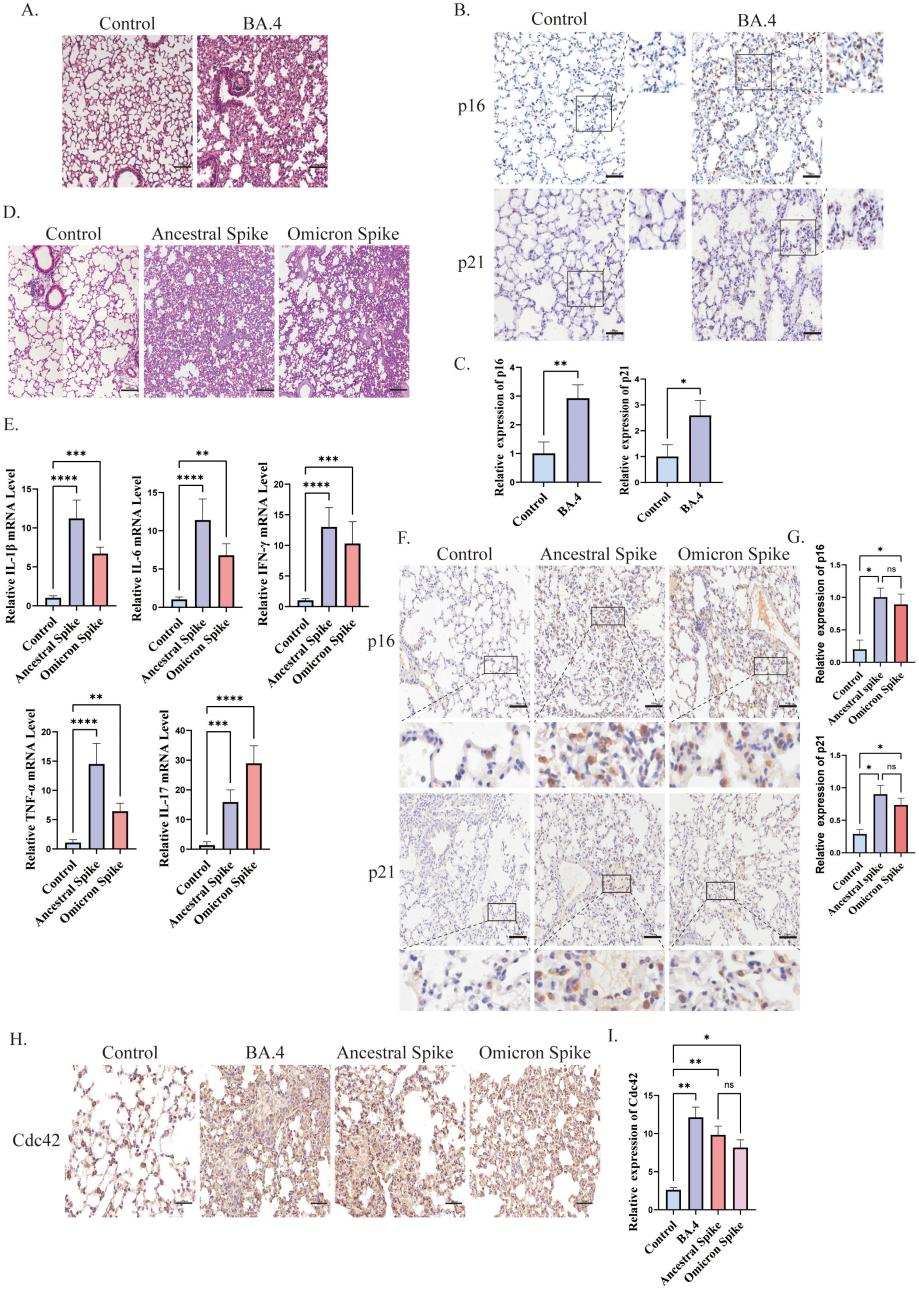
Spike protein accelerates the senescence of alveolar cells. **(A)** H&E staining of lung injury in mice induced by SARS-CoV-2 Omicron BA.4, bar = 100μm; **(B)** IHC staining of p16 and p21 and **(C)** quantitative analysis of p16 and p21 in lung tissue after SARS-CoV-2 Omicron BA.4 infection, bar = 50μm; **(D)** H&E staining of lung injury in mice induced by spike protein, bar = 100μm; **(E)** mRNA expression of IL-1β, IL-6, TNF-α, INF-γ, and IL-17 in lung tissues of K18-hACE2 mice stimulated by spike protein; **(F)** IHC staining and **(G)** quantitative analysis of p16 and p21 in lung tissue of mice stimulated by spike protein, bar = 50μm; **(H)** IHC staining and **(I)** quantitative analysis of Cdc42 in lung tissue of mice under different treatments, bar = 50μm. Data represent the mean ± SD (n=6). *p<0.05, **p<0.01, ***p<0.001.

Cellular senescence has been implicated in the poor clinical outcomes of patients with COVID-19 (Paschalaki et al., 2021). In our study, we induced lung injury in mice using SARS-CoV-2 Omicron BA.4 (**Figure 1A**) and identified senescent cells through immunohistochemical staining. Our results demonstrated an increase in the expression of senescence-related markers, p16, and p21, in the lungs of K18-hACE2 mice four days after infection with SARS-CoV-2 Omicron BA.4 (**Figures 1B, C**), indicating a potential role of novel coronaviruses in triggering cellular senescence.

To further investigate the role of the SARS-CoV-2 spike protein in promoting cellular senescence, we administered the spike protein to K18-hACE2 mice via the trachea. Subsequently, we observed lung injury and evaluated the senescence of alveolar epithelial cells. Given the emergence of several SARS-CoV-2 variants with heightened transmissibility and immune evasion, such as the Omicron variants, these have become dominant globally and have led to increased morbidity (He et al., 2023). Therefore, in this study, we focused on the potential mechanisms of cellular senescence induced by both ancestral and omicron spike variants. Post-treatment with the spike protein, the lungs of mice displayed signs of damage and elevated inflammation levels. Consistent with other reports (Sievers et al., 2022; Wolter et al., 2022), histological examination of ancestral spike-stimulated mice lungs revealed prominent pathological changes in the alveoli, including alveolar wall collapse and infiltration of inflammatory cells. Conversely, Omicron spike-stimulated mice exhibited less severe pathological changes and inflammation (**Figures 1D, E**). Additionally, an increased senescence phenotype was observed compared with that in the control group (**Figures 1F, G**).

The relationship between Cdc42 and aging is well-established, thus, we also examined its expression. We observed upregulation of Cdc42 expression post-infection with SARS-CoV-2 Omicron BA.4 or following spike protein stimulation (**Figures 1H, I**), indicating that the spike protein induces senescence in alveolar epithelial cells in mice, and Cdc42 may be involved in this process.

### 3.2 ML141 alleviates spike protein-induced cellular senescence

**Figure 2:**
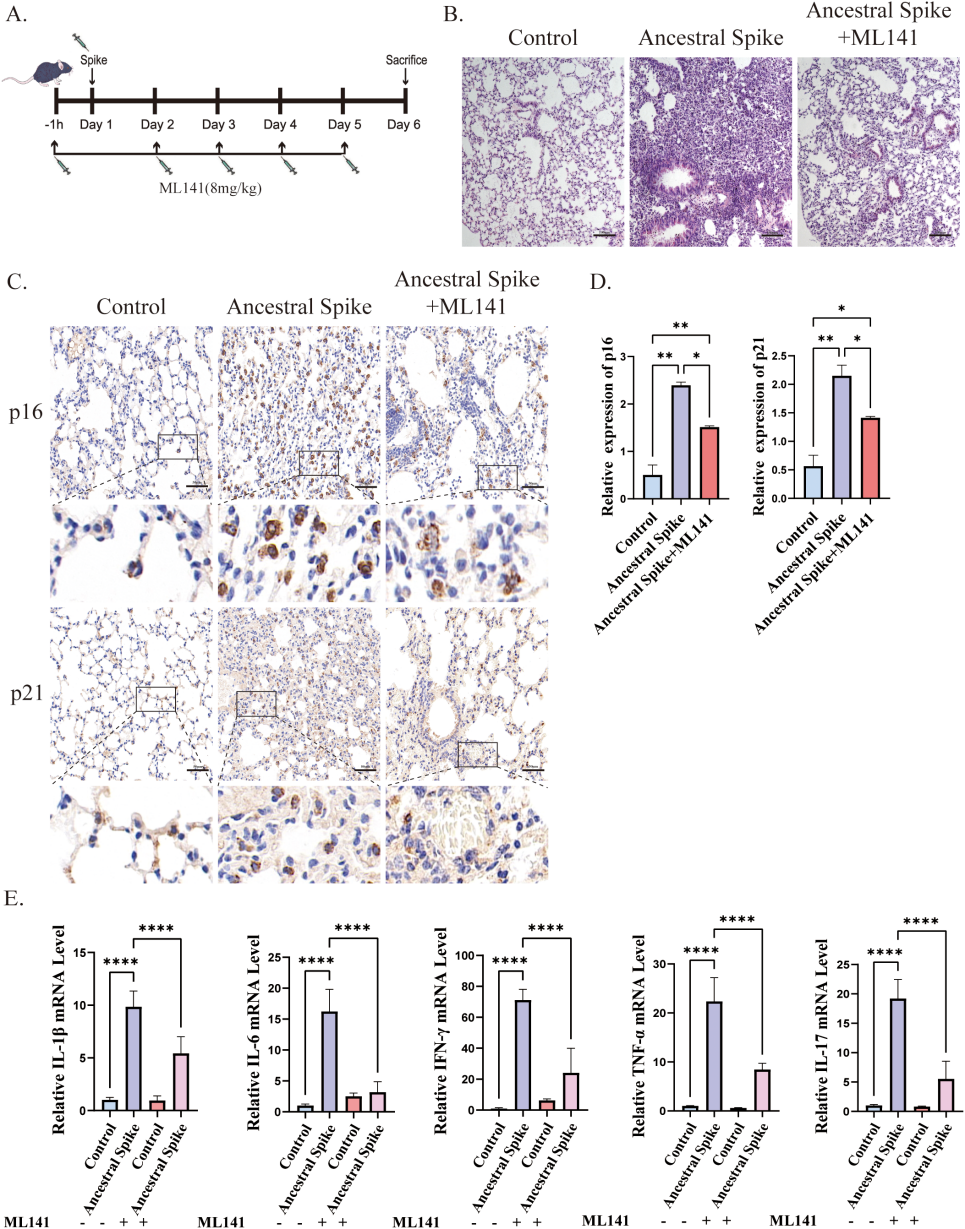
ML141 attenuates spike protein-induced senescence-related phenotypes in mice lung tissues. **(A)** Diagram showing the experimental design for animal treatment; **(B)** H&E staining of mice lung injury induced by spike protein was alleviated by ML141, bar = 100 μm; **(C)** IHC staining of p16 and p21 in mouse lung tissue and **(D)** quantitative analysis, bar = 50μm; **(E)** mRNA expression of inflammatory factors in mouse lung. Data represent the mean ± SD (n=6). *p<0.05, **p<0.01 and ***p<0.001.

To investigate the role of Cdc42 in initiating cellular senescence triggered by the SARS-CoV-2 spike protein, we employed the Cdc42 inhibitor ML141 and assessed senescent cells status in mouse lung tissues post-spike protein stimulation. The experimental flow chart is depicted in **Figure 2A**.

Immunohistochemical analysis revealed that ML141 prevented the spike protein-induced increase in the number of senescent cells in the lungs, primarily manifested by decreased expression of p16 and p21, however, the number of senescent cells remained higher than that in the control group after ML141 treatment (**Figures 2C, D**). Additionally, ML141 administration resulted in a reduction in the levels of inflammatory factors and contributed to the alleviation of lung injury in mice, but conditions did not return to normal compared with that in the control group (**Figures 2B, E**).

### 3.3 Cdc42 expression is significantly elevated in the senescent cells induced by spike protein

**Figure 3:**
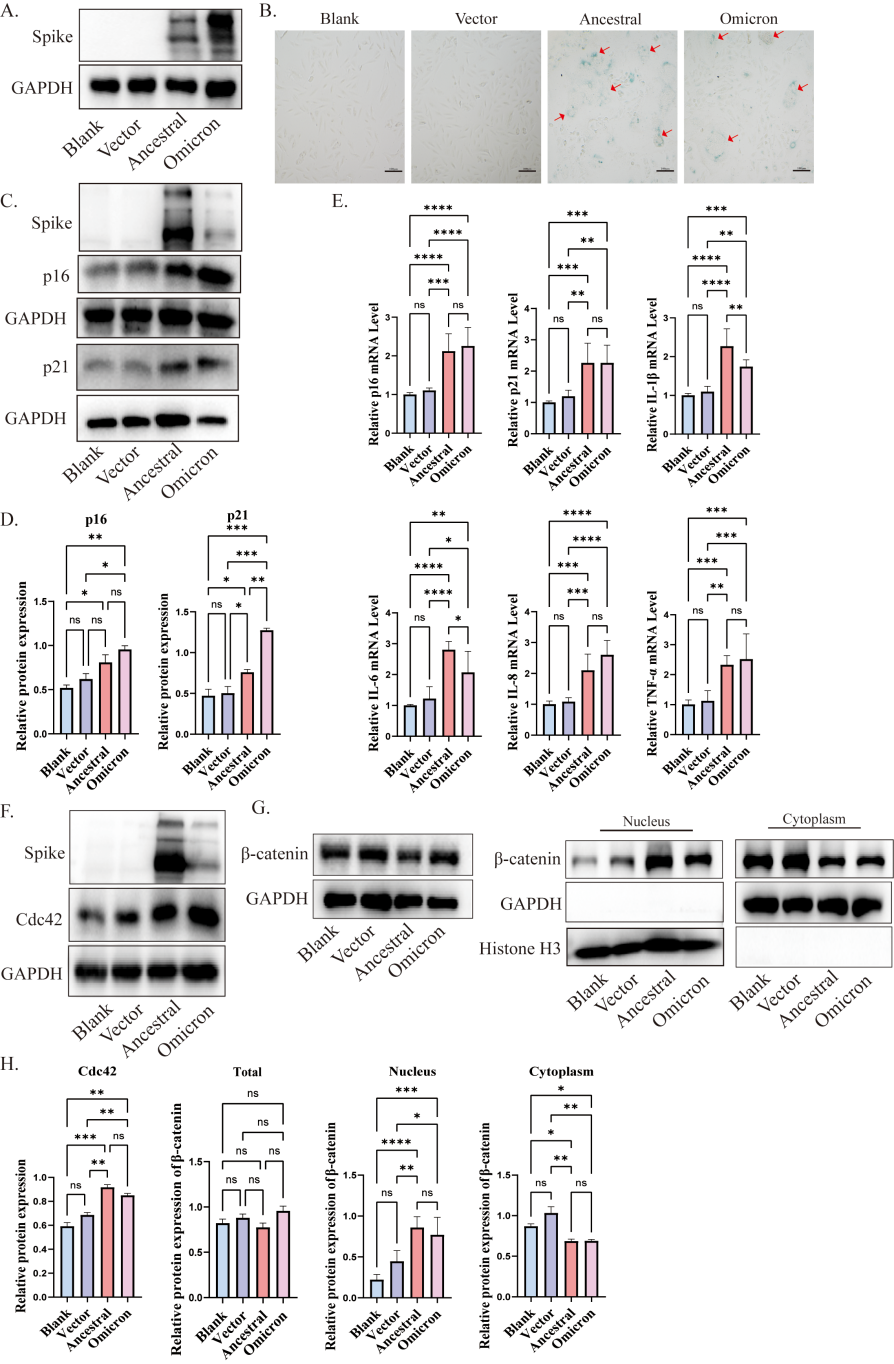
Cdc42 expression increases in senescent cells induced by spike protein. **(A)** Expression of spike protein in ACE2/A549 cells transfected with plasmids for 72 h; **(B)** SA-β-Gal staining of ACE2/A549 cells in different treatment groups and the red arrow indicates spike protein-mediated membrane fusion, bar =100μm; **(C)** Western blot analysis of senescence indicators p16, p21 in ACE2/A549 cells under different treatment factors and **(D)** quantitative analysis; **(E)** mRNA levels of p16, p21, and SASP in ACE2/A549 cells in different groups; **(F)** Western blot analysis of Cdc42 expression and level of β-catenin in the whole cells under different treatments; **(G)** Western blot analysis of β-catenin in cytoplasm and nucleus and **(H)** quantitative analysis. Data represent the mean ± SD (n=3). *p<0.05, ** p<0.01, ***p<0.001.

In our *in vivo* experiments, we observed that the spike protein promotes lung aging in mice. To further explore the role of the spike protein in aging, we incubated spike protein exogenously and transfected ACE2/A549 cells with different plasmids: pcDNA-SARS-CoV-2 S (Ancestral), pcDNA3.1-Spike-Omicron, and pcDNA3.1 empty vector as a control. Subsequently 72h post-transfection, we confirmed the expression of cellular spike protein (**Figure 3A**).

SA-β-Gal, a lysosomal hydrolase and classic marker of cellular senescence (Esposito et al., 2024). Following transfection with SARS-CoV-2 ancestral-spike and omicron-spike plasmids, we observed an increase in the number of SA-β-Gal-positive cells, along with the formation of giant fusion cells induced by the spike protein (**Figure 3B**). Consistent with the experimental results *in vivo*, both p16 and p21 were elevated under the stimulation of spike protein, and the expression of p16 and p21 induced by omicron spike seems to be higher than that induced by ancestral (**Figures 3C, D**), and the expression of some SASP factors was increased compared to the control group (**Figure 3E**). Cdc42 expression, consistent with the *in vivo* results, increased after spike transfection (**Figures 3F, H**). Moreover, treatment of ACE2/A549 cells with purified spike protein for 96 hours enhanced protein levels of p16, p21, and Cdc42, indicating that spike protein incubation could accelerate cellular senescence, potentially mediated by Cdc42 (**Supplementary Figure 1B**).

β-Catenin, a downstream effector of WNT signals and closely related to cellular senescence (Zhou et al., 2015). Previous research has shown that WNT/β-catenin activity is increased in the type II alveolar epithelial cells of aged mice compared to young mice, and prolonged activation of WNT/β-catenin signaling accelerates cellular senescence (Lehmann et al., 2020). β-catenin is a substrate of GSK3β, in the resting state, GSK3β and CKI could phosphorylate β-catenin, triggering its destabilization and degradation to maintain the balance of β-catenin in the cytosol/nucleus. We observed increased phosphorylation of GSK3β, suggesting decreased GSK3β activity (**Supplementary Figures 1A, C**). Compared with control cells, following spike protein stimulation or overexpression, β-catenin translocated to the nuclear compartment, indicating activation of the WNT/β-catenin pathway (**Figures 3G, H**, **Supplementary Figure 1C**). Collectively, our findings suggest that spike protein induces cellular senescence, potentially mediated by regulation of Cdc42 expression and β-catenin translocation to the nucleus.

### 3.4 Inhibition of Cdc42 alleviates senescence-associated phenotypes promoted by spike protein

**Figure 4:**
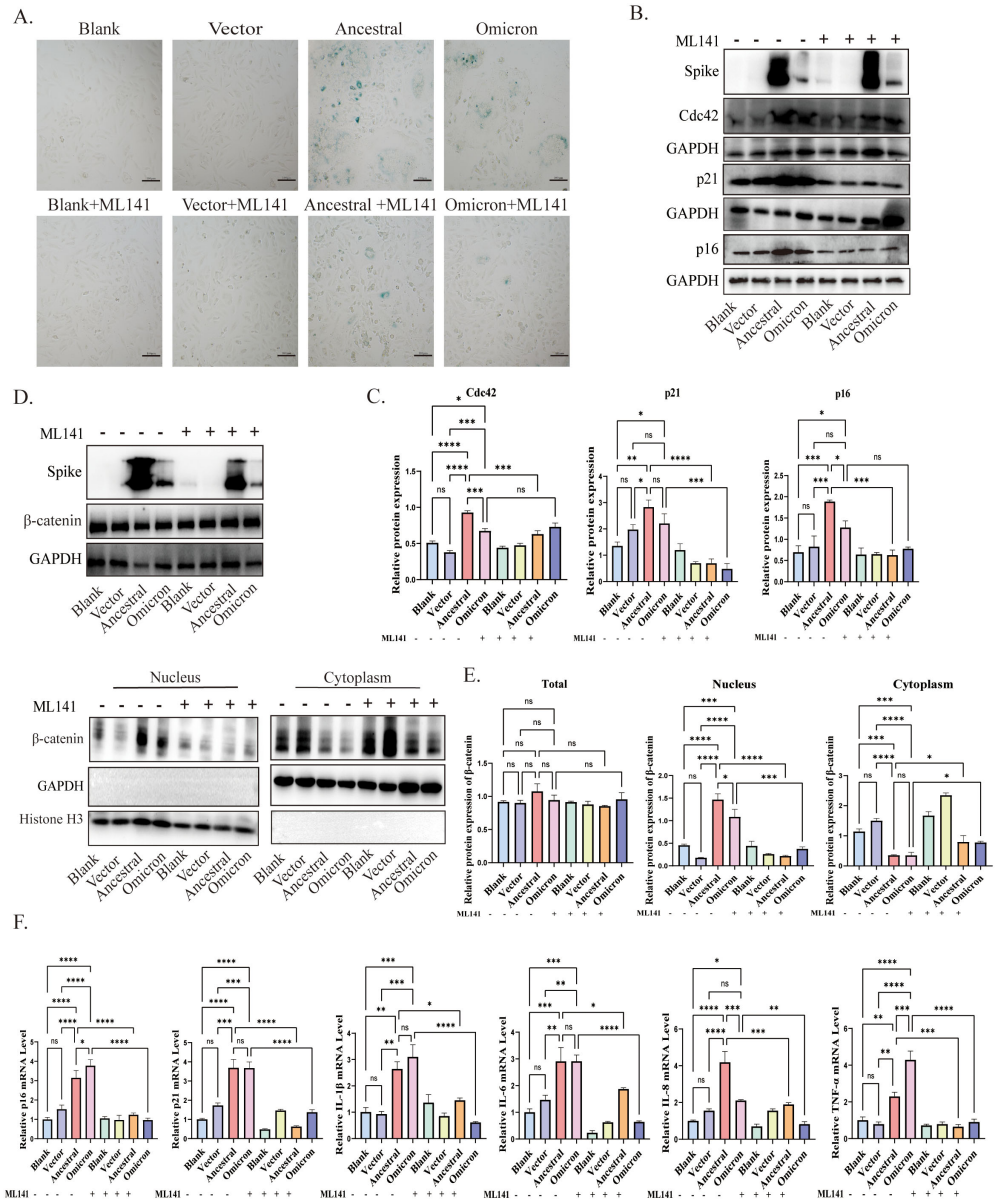
Cdc42 inhibition attenuates spike protein-induced senescence phenotype. **(A)** SA-β-Gal staining of ACE2/A549 cells in different treatment groups, bar = 100μm; **(B)** Western blot detection of Cdc42, p16, and p21 expression in ACE2/A549 cells under different treatments and **(C)** quantitative analysis; **(D)** Western blot detection of the whole cell, cytoplasmic and nuclear expression levels of β-catenin and **(E)** quantitative analysis; **(F)** mRNA levels of p16, p21, and SASP in different groups. Data represent the mean ± SD (n=3). *p<0.05, ** p<0.01, ***p<0.001.

To investigate the contribution of Cdc42 upregulation to spike-induced senescence, we pretreated ACE2/A549 cells, which overexpressed or incubated with spike protein, with the Cdc42 inhibitor ML141. SA-β-Gal staining assessed cellular senescence, revealing that ML141 treatment effectively blocked the spike-mediated increase in the number of senescent cells (**Figure 4A**). Furthermore, the upregulation of p16, p21, and SASP, induced by spike protein, was abolished by ML141 treatment, and for the other inflammatory factors, there were no significant differences between ancestral and omicron, but it seems to be a higher TNF-α level of omicron induced than that in ancestral, while there was no significant change in Cdc42 (**Figures 4B, C, F**). Besides, similar results were obtained in the ACE2/A549 cells groups with exogenous spike protein incubation (**Supplementary Figure 2B**). Compared with the untreated ML141 groups, ML141 reduced the phosphorylation of GSK3β and restored its activity, correspondingly, ML141 significantly inhibited the spike-induced localization of β-catenin to the nucleus (**Figures 4D, E**, **Supplementary Figure 2A, C**). In order to further clarify the role of Cdc42 in regulating cellular senescence, we knockdown Cdc42 by siRNA. Consistent with the effect of using ML141, Cdc42 knockdown resulted in down-regulation of both p16 and p21 expression (**Supplementary Figure 3A**). Meanwhile, the transfecting cells with Cdc42 siRNA abolished GSK3β phosphorylation and reversed the spike protein incubation-induced increase in β-catenin nuclear translocation (**Supplementary Figures 3B, C**).

These findings provide evidence that Cdc42 contributes to cellular senescence triggered by the spike protein. The inhibitory effects of Cdc42 are mainly manifested as the reduction of age-related phenotypes and the inhibition of β-catenin nuclear translocation, highlighting the therapeutic potential of targeting Cdc42 in mitigating spike protein-induced senescence.

### 3.5 Cdc42 drives spike-induced cellular senescence by promoting β-catenin translocation to the nucleus

Our results confirm the central role of Cdc42 in spike protein-mediated cellular senescence. To investigate whether this effect is mediated through the activation of the WNT/β-catenin signaling pathway, we treated ACE2/A549 cells with the WNT/β-catenin pathway inhibitor, KYA1797K, followed by transfection with plasmids.

**Figure 5:**
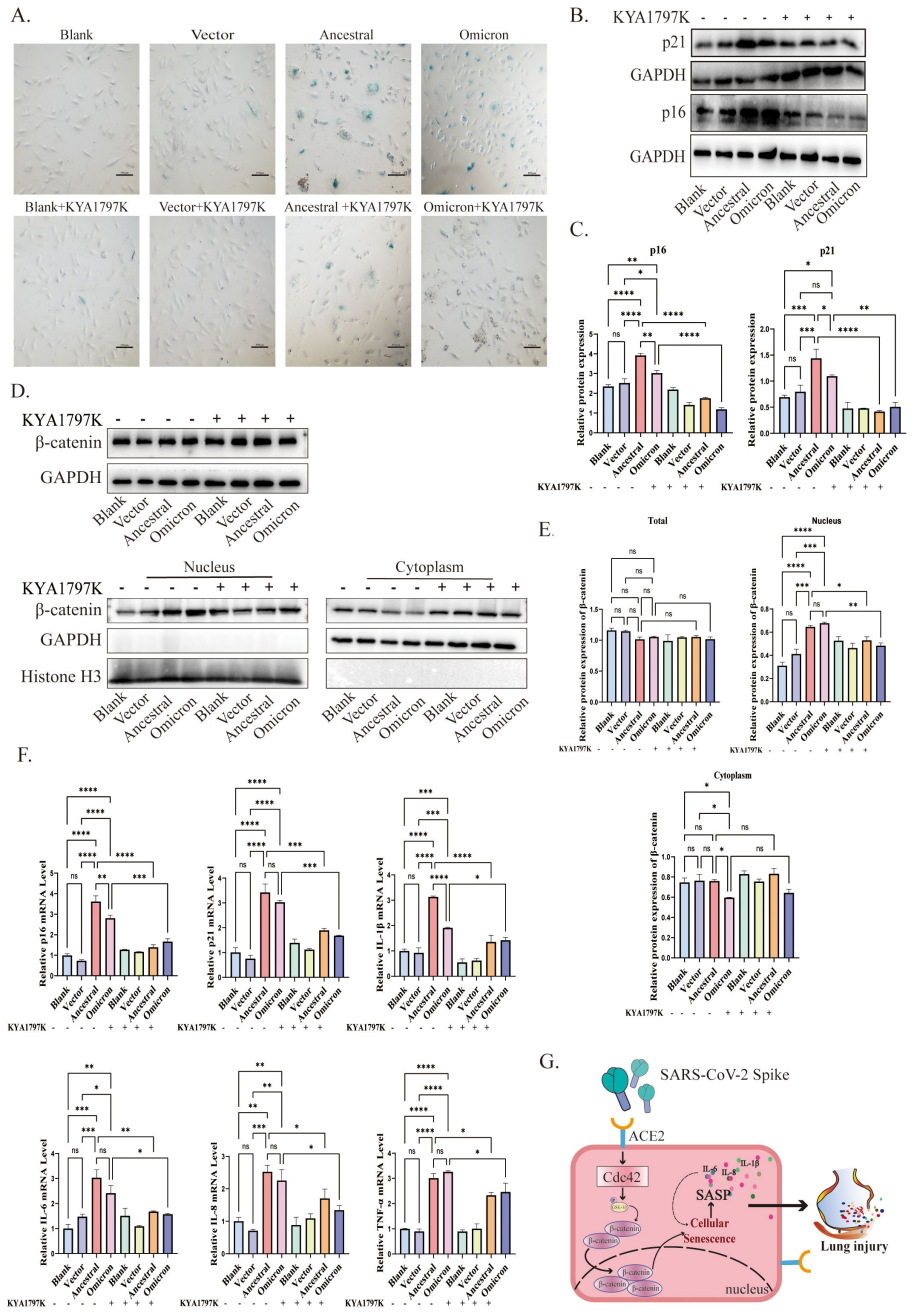
Cdc42 drives senescence by promoting β-catenin translocation to the nucleus. **(A)** SA-β-Gal staining of ACE2/A549 cells in different groups, bar = 100μm; **(B)** Protein levels of p16, p21, and **(C)** quantitative analysis in different treatments; **(D)** Protein levels of β-catenin and **(E)** quantitative analysis in different treatment groups; **(F)** mRNA levels of SASP in different groups; **(G)** Schematic of Cdc42 involved in the spike-induced cellular senescence by promoting β-catenin translocation to the nucleus. Data represent the mean ± SD (n=3). *p<0.05, ** p<0.01, ***p<0.001.

The application of KYA1797K significantly influenced cellular senescence, as evidenced by the decreased number of SA-β-Gal-positive cells compared to groups expressing spike protein alone (**Figure 5A**). Furthermore, upon treatment with KYA1797K, western blot analysis revealed a downregulation of p16 and p21 (**Figures 5B, C**). We also examined the WNT/β-catenin pathway, spike protein overexpression resulted in reduced β-catenin translocation into the nucleus (**Figures 5D, E**). Additionally, the mRNA levels of SASP factors decreased in response to KYA1797K (**Figure 5F**). These findings highlight the impact of the interaction between Cdc42 and the WNT/β-catenin pathway in spike protein-induced cellular senescence.

## 4 Discussion

Aging is a major risk factor for severe COVID-19, with older individuals being more susceptible to worsened symptoms and outcomes (D’Agnillo et al., 2021; Santesmasses et al., 2020). This association may be attributed to the accumulation of senescent cells during aging. Alongside naturally occurring senescent cells, SARS-CoV-2 infection can induce cellular senescence, further exacerbating the accumulation of these cells. When the burden of senescent cells surpasses the capacity of the immune system to eliminate them, they release SASPs, provoking an inflammatory response. This chronic inflammation hampers tissue proliferation and repair, exacerbating disease progression. Several studies have highlighted the potential role of senescent cells in intensifying the immune response against SARS-CoV-2 (Blagosklonny, 2020; Kirkland and Tchkonia, 2020; Malavolta et al., 2020; Nehme et al., 2020). Thus, comprehending the connection between cellular senescence and COVID-19 pathogenesis is crucial for developing therapeutic and preventive strategies against its sequelae.

Senescence is a cellular response that occurs in the presence of various internal and external stress signals. In this study, we investigated the role of the spike protein in triggering cellular senescence. In mice lung tissues exposed to the spike protein, we observed an increase in the number of cells positive for the senescence markers p16 and p21, with ancestral spike-induced cell aging appearing more severe than omicron. Moreover, compared with the ancestral spike, omicron induced substantially attenuated lung pathology with downregulation of proinflammatory cytokines, consistent with the decreased pathogenicity of omicron that has been reported (Chan et al., 2022; Shuai et al., 2022; Tamura et al., 2023), and may even relate to the weaker ability of omicron to induce cellular senescence. Notably, we also noticed an upregulation in the expression of Cdc42, a protein associated with cellular processes.

Cdc42 is an evolutionarily ancient protein widely expressed and involved in fundamental cellular functions. Numerous studies have highlighted its role in the development and progression of age-related pathologies, including neurodegenerative diseases (Stankiewicz and Linseman, 2014), cardiovascular disease (Florian et al., 2017; Hirano et al., 2008), and degenerative joint diseases (Suzuki et al., 2015). For example, in Alzheimer’s disease, there is a significant upregulation of Cdc42 activity (Chen-Plotkin et al., 2009; Zhu et al., 2000). Additionally, while elevated Cdc42 activity has been associated with the senescence of intestinal stem cells, its inhibition enhances the regenerative capacity of the stem cells (Castillo-Azofeifa et al., 2023). Senolytics, which selectively eliminate senescent cells by inducing apoptosis, have shown promise in delaying, preventing, and even reversing the aging process, thereby extending lifespan (Lagoumtzi and Chondrogianni, 2021). Studies focusing on Cdc42 inhibition have demonstrated significant therapeutic and anti-aging effects in aged mice and models of aging-related diseases (Amoah et al., 2022; Florian et al., 2020). Based on these findings, we infer that Cdc42 plays a crucial role in spike protein-induced cellular senescence. For inflammation, inhibition of Cdc42 reduced cytokine secretion during TNF-α-induced inflammation, and also had an effect on cytokine gene transcription. Additionally, other reports showed that Rho proteins are needed for cytokine activation of NF-κB for proinflammatory signaling and it was reported that Cdc42 could promote the release of IL-1β by activating IQGAP1 (Bahia et al., 2020; Rowayna and Gary, 2024; Yun et al., 2022). Notably, however, Takashi K et al. suggest that inhibition of Cdc42 signaling had a much weaker influence on acute inflammation than chronic inflammation, which supports the notion that the Cdc42-dependent proinflammatory pathway is specifically activated by senescence-associated stimuli (Takashi K et al., 2014). Inhibition of CDC42, particularly using Cdc42 inhibitors as senolytic-associated drugs could potentially block spike protein-induced cellular senescence, alleviating the inflammatory response and mitigating the disease progression associated with senescent cells.

β-catenin is a crucial molecule involved in the classical WNT signaling pathway. Under normal conditions, the WNT pathway is inactive. However, abnormal activation of the pathway leads to the inhibition of β-catenin phosphorylation and ubiquitination, leading to an increase in free cytoplasmic β-catenin levels. This elevated β-catenin translocates into the nucleus, activating downstream WNT target genes (Angers and Moon, 2009; Vallee et al., 2021). Recent evidence suggests that classical WNT/β-catenin pathway activation is associated with inflammation and cytokine storms in COVID-19 patients (Choi et al., 2020; Vallee et al., 2021). Additionally, the WNT/β-catenin signaling pathway has been implicated in cellular senescence processes such as intervertebral disc degeneration, senescence of type II alveolar epithelial cells promoting pulmonary fibrosis, and renal tubular senescence (Chen et al., 2019; Gong et al., 2021; Lehmann et al., 2020; Yang et al., 2022). Furthermore, the relationship between Cdc42 and β-catenin has been established, with Cdc42 being involved in cell proliferation, migration, and differentiation through the activation of WNT/β-catenin signaling (Han et al., 2017; Wu et al., 2006). Cdc42 is known to initiate p-PKC*ζ*/pGSK3β signaling, which is involved in renal fibrosis by inhibiting p-β-catenin and upregulating β-catenin (Hu et al., 2024). Consistently, we observed that Cdc42 regulates β-catenin, with Cdc42 inhibition increasing its degradation and reducing its entry into the nucleus. It has been reported that the increase of β-catenin nuclear translocation can induce cellular senescence by activating the p53/p21 signaling pathway, and in our study, spike protein was found to promote the up-regulation of p21 expression (Gu et al., 2014). Furthermore, p16 was found to colocalize with β-catenin to regulate the cellular aging process, indicating that these signals are closely related (Meng et al., 2022).

In our study, we observed that the spike protein induces increased translocation of β-catenin into the nucleus, activating the WNT/β-catenin pathway. However, Cdc42 knockdown and the use of ML141, a Cdc42 inhibitor, and KYA1797K, a WNT/β-catenin pathway inhibitor, can block the translocation of cytoplasmic β-catenin into the nucleus, thus alleviating the senescence-associated phenotype induced by SARS-CoV-2 infection.

In conclusion, our study findings confirm that the spike protein of SARS-CoV-2 promotes β-catenin translocation into the nucleus by upregulating Cdc42. This activates the WNT/β-catenin pathway, inducing cellular senescence (**Figure 5G**). By inhibiting Cdc42 activity, we observed decreased spike-induced cellular senescence and lung injury alleviation in mice lung tissues. These findings suggest targeting Cdc42 as a therapeutic strategy may mitigate the detrimental effects of spike protein-induced cellular senescence associated with SARS-CoV-2 infection and the lack of validation of possible pathways by which Cdc42 regulates related inflammatory factors is a limitation of this study. Further study is warranted to explore the precise mechanisms underlying the interplay between Cdc42, β-catenin, spike protein-mediated cellular senescence, and inflammatory response, and evaluate the therapeutic implications of targeting Cdc42 in the context of COVID-19 and other age-related diseases.

## Data Availability

The raw data supporting the conclusions of this article will be made available by the authors, without undue reservation.
