# Supplementary material for: Cdc42 improve SARS-CoV-2 spike protein-induced cellular senescence through activating of Wnt/β-Catenin signaling pathway
Source: Front Cell Infect Microbiol. 2024 Nov 4;14:1449423. doi: 10.3389/fcimb.2024.1449423 (PMC11570593; doi:10.3389/fcimb.2024.1449423)
Supplement: Supplementary file 1 [file DataSheet1.docx]

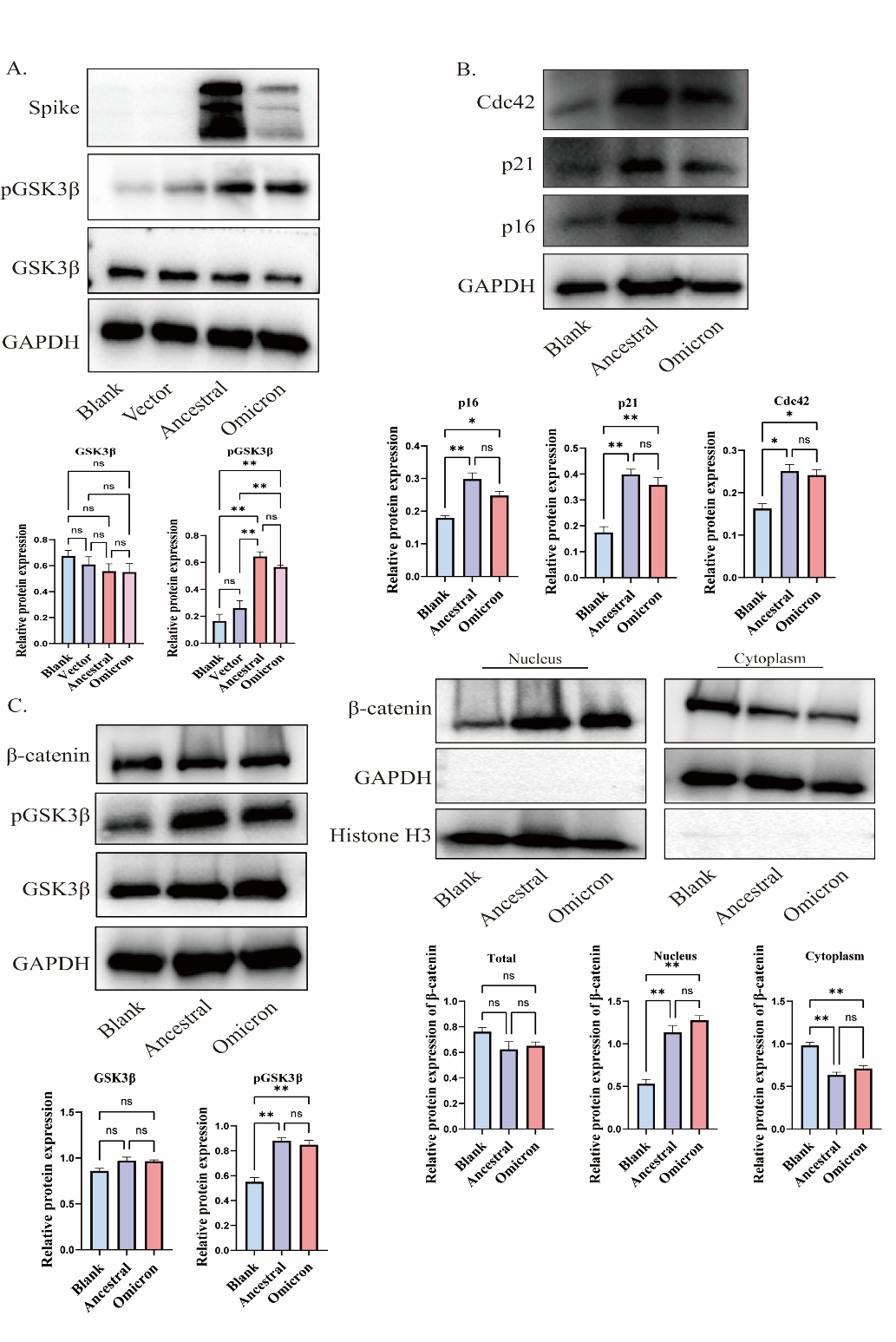


Supplementary Figure 1. Spike protein activates the WNT/β-catenin pathway. (A)Western blot detection of GSK3β, pGSK3β, and spike protein**;** (B) Protein levels of Cdc42 and cellular senescence related indicators of spike protein incubation; (C) Protein levels of WNT/β-catenin pathway related indicators after co-incubation with spike protein. Data represent the mean ± SD (n=3). *p<0.05, ** p<0.01, ***p<0.001.


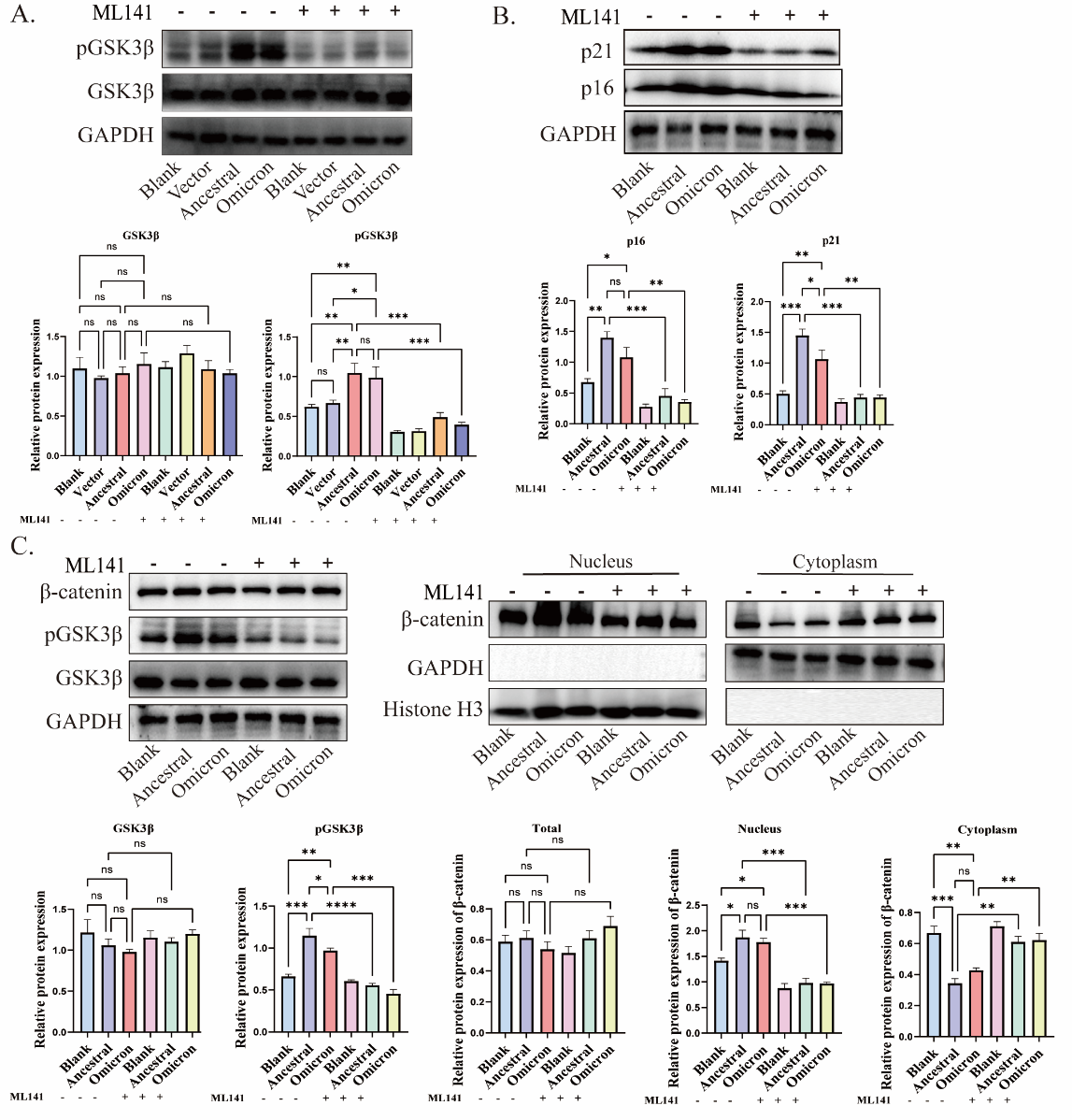


Supplementary Figure 2. Cdc42 inhibition attenuates spike protein-induced β-catenin translocation to the nucleus. (A) The protein level of GSK3β, pGSK3β with or without ML141 treatment; (B) Western blot detection of p16 and p21 of spike protein incubation with or without ML141 treatment; (C) The protein level of WNT/β-catenin pathway related indicators after co-incubation with spike protein with or without ML141 treatment. Data represent the mean ± SD (n=3). *p<0.05, ** p<0.01, ***p<0.001.


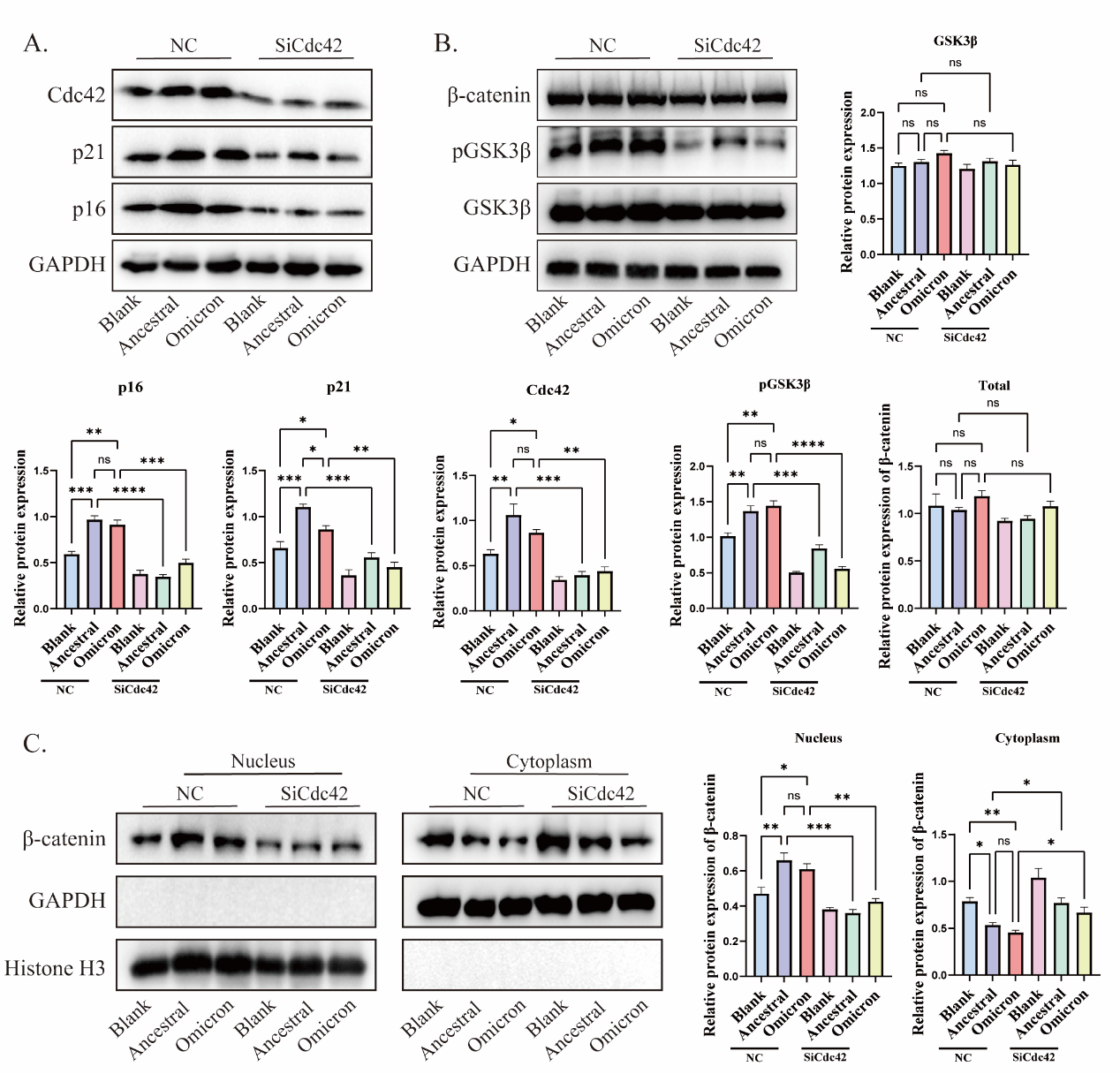


Supplementary Figure 3. Cdc42 knockdown attenuates spike protein-induced activation of the WNT/β-catenin signaling pathway. (A) The protein level of Cdc42, p16, and p21 in ACE2/A549 cells with or without Cdc42 knockdown; (B) Western blot detection of β-catenin, pGSK3β, and GSK3β with or without Cdc42 knockdown; (C) Western blot detection of β-catenin in nucleus and cytoplasm with or without Cdc42 knockdown. Data represent the mean ± SD (n=3). *p<0.05, ** p<0.01, ***p<0.001.
